# Cost-Effectiveness of Surveillance after Metastasectomy of Stage IV Colorectal Cancer

**DOI:** 10.3390/cancers15164121

**Published:** 2023-08-16

**Authors:** Philip Q. Ding, Flora Au, Winson Y. Cheung, Steven J. Heitman, Richard Lee-Ying

**Affiliations:** 1 Oncology Outcomes Program, Department of Oncology, University of Calgary, Calgary, AB T2N 4Z6, Canada; 2 Faculty of Medicine & Dentistry, University of Alberta, Edmonton, AB T6G 2R3, Canada; 3 Department of Medicine, Cumming School of Medicine, University of Calgary, Calgary, AB T2N 4Z6, Canada; 4 Department of Oncology, Cumming School of Medicine, University of Calgary, Calgary, AB T2N 4N2, Canada; 5 Department of Community Health Sciences, Cumming School of Medicine, University of Calgary, Calgary, AB T2N 4Z6, Canada

**Keywords:** colorectal cancer, metastatic resection, surveillance, cost-effectiveness, health economics, decision analysis, Markov model

## Abstract

**Simple Summary:**

Up to 65% of patients with metastatic colorectal cancer experience disease recurrence following curative-intent metastasectomy. Certain oncology organizations recommend surveillance to detect recurrent disease at an early phase to allow for the greatest benefit from additional treatment. Surveillance programs may involve colonoscopy, physical examination, carcinoembryonic antigen testing, and computed tomography at regular intervals. Even though surveillance can be highly effective, there is little evidence for its use in metastatic colorectal cancer and no clear consensus on the best strategy. We used decision analysis and a population-based cohort to evaluate the cost-effectiveness of various surveillance strategies following curative-intent metastasectomy in patients with stage IV colorectal cancer. Our results show that surveillance with clinic visits and investigations every 12 months for 5 years would be cost-effective in a Canadian context. These findings demonstrate the utility of economic analysis for guiding the management of stage IV colorectal cancer after metastasectomy.

**Abstract:**

Surveillance of stage IV colorectal cancer (CRC) after curative-intent metastasectomy can be effective for detecting asymptomatic recurrence. Guidelines for various forms of surveillance exist but are supported by limited evidence. We aimed to determine the most cost-effective strategy for surveillance following curative-intent metastasectomy of stage IV CRC. We performed a decision analysis to compare four active surveillance strategies involving clinic visits and investigations elicited from National Comprehensive Cancer Network (NCCN) recommendations. Markov model inputs included data from a population-based cohort and literature-derived costs, utilities, and probabilities. The primary outcomes were costs (2021 Canadian dollars) and quality-adjusted life years (QALYs) gained. Over a 10-year base-case time horizon, surveillance with follow-ups every 12 months for 5 years was most economically favourable at a willingness-to-pay threshold of CAD 50,000 per QALY. These patterns were generally robust in the sensitivity analysis. A more intensive surveillance strategy was only favourable with a much higher willingness-to-pay threshold of approximately CAD 425,000 per QALY, with follow-ups every 3 months for 2 years then every 12 months for 3 additional years. Our findings are consistent with NCCN guidelines and justify the need for additional research to determine the impact of surveillance on CRC outcomes.

## 1. Introduction

Colorectal cancer (CRC) is the third most commonly diagnosed cancer and the second leading cause of cancer-related death worldwide [1]. Although CRC mortality rates in high-income countries are among the highest in the world, incidence rates are declining [2]. Improvements in CRC outcomes are linked to best practices in screening and advances in systemic therapy and surgical management [3]. Nevertheless, recurrent disease occurs in ~30% of patients with stage I-III CRC and up to 65% of patients with resected stage IV cancer [4,5,6,7,8]. Thus, patients enter surveillance programs with the principal goal of detecting recurrent disease, new primary cancers, and metastases at an early, asymptomatic phase for the greatest potential benefit from further intervention including metastatic resection.

Approximately 10–20% of patients with de novo stage IV or recurrent metastatic CRC are eligible for curative-intent metastasectomy, a procedure associated with a potential for improved long-term outcomes and, sometimes, a cure [9,10]. The location and extent of disease are key determinants in the resectability of patients with hepatic or pulmonary CRC metastases [11,12]. Early detection of recurrence through surveillance increases the likelihood of further curative-intent treatment, such as repeat metastasectomy [7,8,13,14]. In a retrospective analysis of 257 Canadian patients who had curative-intent metastasectomy of stage IV CRC, our group found that patients with recurrent disease detected while asymptomatic were 4.6-times more likely to undergo repeat metastasectomy compared to those with symptomatic recurrence [15]. Moreover, surveillance and repeat metastasectomy were both independently associated with greater overall survival in multivariable analyses. 

Intensive surveillance strategies after curative-intent resection of stage I-III CRC are advised by most major oncology organizations [16,17,18,19,20,21,22] and have been shown to improve outcomes following CRC recurrence [7,9,23,24]. However, surveillance schemes for patients with stage IV CRC treated with curative-intent metastasectomy are less clear, with existing studies limited to patients with CRC liver metastases [6,19,22,25,26,27,28,29,30]. Among the most extensive guidelines are those of the National Comprehensive Cancer Network (NCCN) advocating physical examination, carcinoembryonic antigen (CEA) testing, and computed tomography (CT) every 3–6 months for the first 2 years, and then every 6–12 months for an additional 3 years, in addition to colonoscopy in the first 6–12 months and then in 3 years then every 5 years [19]. The economic implications of surveillance among patients having undergone curative-intent metastasectomy is not well described and there is no clear consensus on the most cost-effective approach [28,31]. 

To address these knowledge gaps, we performed a full-scale economic evaluation of potential surveillance strategies after curative-intent metastasectomy of stage IV CRC in a Canadian context. 

## 2. Methods

### 2.1. Overview

We constructed a decision analytic model to compare various modalities and intervals of surveillance based on NCCN guidelines [32]. Costs, utilities, and probabilities were derived from the literature and population-based outcomes observed in a real-world patient cohort from British Columbia, Canada.

We performed a cost-utility analysis among patients who underwent curative-intent metastasectomy of stage IV CRC. We compared the following surveillance schedules for clinic visits, CEA testing, and CT with varying intensity: every 3 months for 2 years and then every 12 months for 3 additional years (“q3/q12”), every 6 months for 5 years (“q6”), every 6 months for 2 years and then every 12 months for 3 additional years (“q6/q12”), and every 12 months for 5 years (“q12”). We assumed that all patients under active surveillance also underwent colonoscopy at 1 year and 4 years [15], assuming most patients presented with de novo metastatic disease. As patients can also present with metachronous metastases and already be on a colonoscopy surveillance schedule every 3–5 years, we did not include a colonoscopy at 9 years, so that at most 2 colonoscopies over a 10-year time horizon would be performed. 

We built a Monte Carlo micro-simulation using a 1-month cycle length and 10-year time horizon, with 10,000 iterations. In the reference case, costs were calculated from a publicly funded healthcare payer perspective. We measured health outcomes in quality-adjusted life-years (QALYs). Outcomes included cost, QALYs, and cost per QALYs/LYs and were half-cycle corrected. We also conducted exploratory analyses using life-years (LYs) as an outcome. We discounted future costs and outcomes at 1.5% annually in accordance with Canadian Agency for Drugs and Technologies in Health (CADTH) guidelines [33]. Incremental analyses (expressed as cost per QALY/LY gained) involved rank ordering all competing strategies by increasing costs after eliminating strategies that were more costly and less effective (i.e., dominated). We performed and reported the results of this economic evaluation according to the Consolidated Health Economic Evaluation Reporting Standards 2022 (CHEERS 2022) [34].

### 2.2. Model

**Figure 1:**
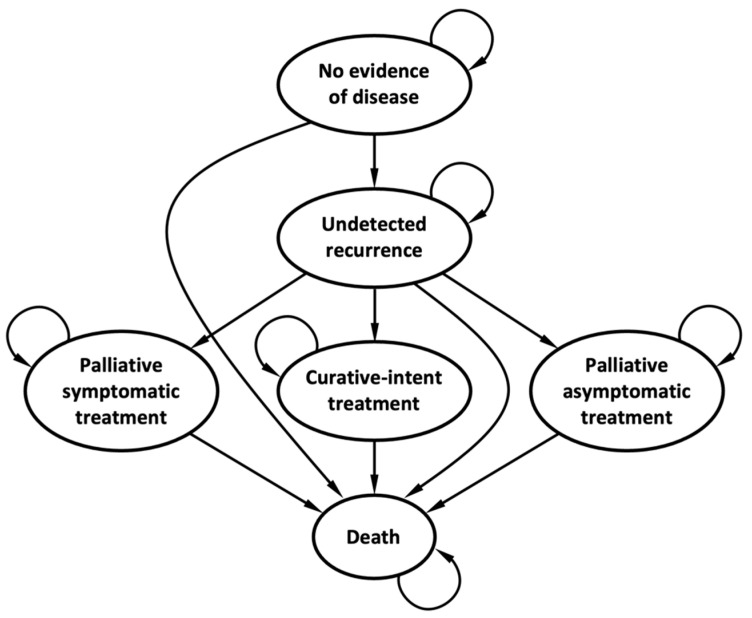
Markov model structure.

We developed a Markov model using decision analysis software (TreeAge Pro 2021, R1.2 Healthcare; Williamstown, MA, USA). All patients were initially assumed to have no evidence of disease following curative-intent metastasectomy for stage IV CRC, from which they could then develop recurrent disease. This recurrence may then be detected once symptoms develop or while undergoing surveillance during an asymptomatic phase. Upon detection, patients can be curatively treated, the likelihood of which is greater for asymptomatic disease, or undergo palliative treatment with either symptomatic or asymptomatic disease. We incorporated several general health states: (1) alive with no evidence of disease, (2) alive with undetected recurrent disease, (3) alive and receiving curative-intent treatment, (4) alive and undergoing palliative treatment for symptomatic disease, (5) alive and receiving palliative treatment for asymptomatic disease, and (6) deceased. Patients receiving curative-intent treatment were assumed to have undergone hepatic metastectomy and 6 months of peri-operative oxaliplatin-based chemotherapy. Palliative treatment consisted of FOLFOX, FOLFIRI, and bevacizumab. Molecular subtypes (e.g., mismatch-repair deficient), more targeted treatment (e.g., immunotherapy, EGFR monoclonal antibodies, and BRAF-inhibitors), and late-lines of treatment (TAS-102 and Regorafenib) were not accounted for, as they were not available during the study reference period. Each month, the patients could remain in the same health state, progress to a subsequent health state, or die (Figure 1).

### 2.3. Model Inputs

We modelled recurrence, repeat resection rates, and survival outcomes from the population-based outcomes of 257 patients who had curative-intent metastasectomy of stage IV CRC in British Columbia, Canada, and subsequently no evidence of disease [15]. These patients were identified from a larger cohort of 2082 stage IV CRC patients diagnosed and treated from 1995–2010 at the British Columbia Cancer Agency Gastrointestinal Cancer Outcomes Unit, which has been previously described [35]. The rate of loss to follow-up from this cohort was estimated to be less than 5%. The most common site of metastatic resection was the liver (65%), followed by the lungs (16%), intra-abdominal disease (14%), and other sites (5%). Intra-abdominal disease included bowel, lymph node, peritoneal, and pelvic metastases. With a median follow-up time of 76.4 months, the recurrence rate was 75.1%. The 5- and 10-year overall survival rates were 25.4% and 3.1% for patients who recurred, and 81.5% and 81.5% for patients who did not recur, respectively [15].

Costs, utilities, and probabilities are presented in Table 1. All costs are in 2021 Canadian dollars, effectiveness in QALYs or LYs, and cost per QALY or LY from the Canadian public payer perspective. 

### 2.4. Scenario and Sensitivity Analyses

We completed one-way and two-way sensitivity analyses to estimate the impact of plausible variations in time to symptomatic recurrence and the probability of repeat curative-intent resection. As the probability of repeat curative-intent resection was derived from observational data, we varied the rates by 25% in the opposite directions for symptomatic and asymptomatic recurrence to approximate a maximum and minimum difference between the observed rates. Using distributional assumptions of the input parameters (Table 1), we also completed a probabilistic sensitivity analysis (PSA) whereby we allowed all variables to change simultaneously through 10,000 Monte Carlo simulations.

## 3. Results

### 3.1. Cost Estimates

**Figure 2:**
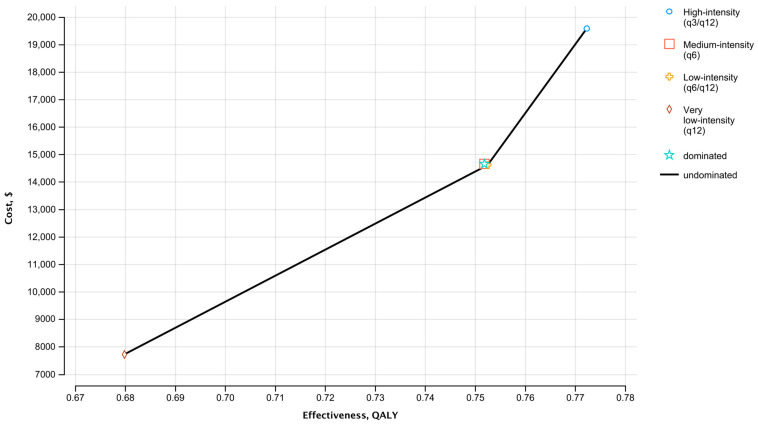
Base case cost-effectiveness frontier comparing four active surveillance strategies with varying intensity of follow-up. QALY, quality-adjusted life year.

We estimated the cost of surveillance for a patient after curative-intent metastasectomy of stage IV CRC to be CAD 19,890 for the q3/q12 strategy, CAD 14,191 for q6, CAD14,175 for q6/q12, and CAD 7707 for q12 (Table 2, Figure 2). Increasing the frequency of surveillance tests led to modest gains in effectiveness, from 0.68 QALYs or 0.90 LYs for the q12 strategy to 0.77 QALYs or 1.01 LYs for the q3/q12 strategy. However, the cost of surveillance and subsequent treatment was high. In both cost-utility and cost-effectiveness analyses, the q6 strategy was eliminated by extended dominance, and the q12 strategy was the most cost-effective surveillance strategy.

**Figure 3:**
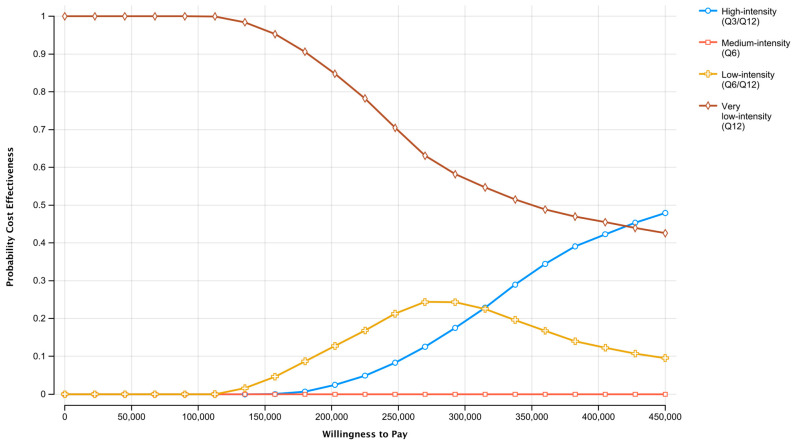
Cost-effectiveness acceptability curves depicting the probability that a surveillance strategy is cost-effective compared to the alternatives, over a range of willingness-to-pay thresholds.

Comparing the active surveillance strategies at willingness-to-pay thresholds of CAD 50,000–100,000 per QALY, the q12 strategy had the highest probability of being most the cost-effective (Figure 3). However, at willingness-to-pay thresholds greater than approximately CAD 425,000 per QALY, the q3/q12 strategy had the highest probability of being cost-effective compared to the other surveillance schemes. The q6 surveillance strategy was the least likely to be cost-effective at any willingness-to-pay threshold.

### 3.2. Probabilistic Sensitivity Analysis

**Figure 4:**
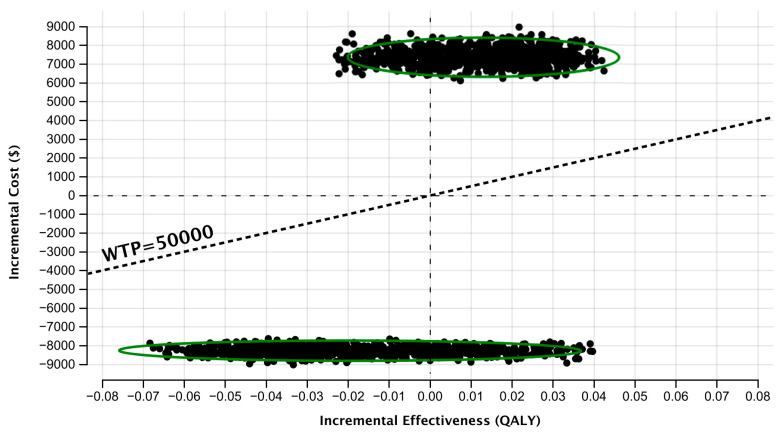
Incremental cost-effectiveness plane obtained through probabilistic sensitivity analysis with 10,000 model simulations for each comparison. Upper cluster, q12 surveillance strategy vs. q6/q12 strategy; lower cluster, q3/q12 strategy vs. q6/q12 strategy. QALY, quality-adjusted life year; WTP, willingness-to-pay.

Figure 4 is an incremental cost-effectiveness plane that depicts the uncertainty in the expected ICERs for the q12 and q3/q12 surveillance strategies compared to the q6/q12 strategy. Of 10,000 iterations per comparison, there was a high degree of certainty that the q12 strategy was less costly than the q6/q12 strategy, while the q3/12 strategy was more costly. The points situated below the diagonal dotted line represent simulations in which the q12 strategy was the cost-effective alternative at a willingness-to-pay threshold of CAD 50,000/QALY.

### 3.3. One-Way and Two-Way Sensitivity Analyses

The results of our base-case analysis were generally robust to uncertainty in the probability of repeat curative-intent resection (Table 3). The q12 surveillance strategy remained favourable in scenarios where the probability of repeat curative resection was altered by 25% in opposite directions, while the q6 strategy remained dominated. Concordantly, the q12 strategy remained the most cost-effective, and q6 was dominated at the 1- and 28-month extremes of time to symptomatic recurrence.

## 4. Discussion

Using a cohort of patients who have undergone curative-intent metastasectomy for stage IV CRC, we modelled and evaluated the cost-effectiveness of different surveillance strategies based on NCCN guidelines. We found that a surveillance scheme with clinic visits, CEA testing, and CT every 12 months for 5 years is likely favoured from a publicly funded healthcare payer perspective at the standard willingness-to-pay threshold of CAD 50,000/QALY [48]. Only at a willingness-to-pay threshold of CAD 425,000/QALY was the higher-intensity strategy with clinic visits and investigations every 3 months for 2 years and then every 12 months for 3 additional years most likely cost-effective. Surveillance with follow-up every 6 months for 5 years was dominated in most scenarios and cannot be recommended routinely.

Active surveillance following metastasectomy is the accepted clinical practice when resources are sufficient [19]. Even though no surveillance is likely a plausible approach, with it associated with not only lower costs but also poorer outcomes, active surveillance of resected stage IV CRC is a cornerstone of effective follow-up care, facilitating effective treatment and survivorship care planning. Therefore, active surveillance should be offered in most settings in the context of shared decision-making [49].

This study focused on surveillance as a package, and although imaging contributed to upfront costs, the downstream costs of managing recurrence were the greatest driver of costs in our model. Further metastasectomy and varying systemic therapy regimens have demonstrated incremental cost-effectiveness in prior studies but when combined with surveillance in our model, their benefits appeared to attenuate [30,50,51].

The methodology used in this study was unique in its use of a population-based cohort to inform patient outcomes. Randomized studies have focused exclusively on the role of perioperative systemic treatments and metastatic resection, not formally on the role of surveillance afterwards [52,53]. The few randomized clinical trials that incorporated metastatic resections have also narrowed the scope to hepatic resection only, while our datasets also allowed for the assessment of surveillance following metastasectomy of extrahepatic sites such as the lung [6,52,53]. The comprehensiveness of our data broadens the generalizability of the study findings. Despite the inherent limitations of using a non-randomized retrospective dataset to inform model outcomes, this study provides valuable insights into the optimal frequency of surveillance testing.

The differences in outcomes were largely driven by the differences in resectability for symptomatic and asymptomatic recurrence. Symptomatic disease was used as a surrogate for repeat resectability since increases in the size, number, and location of metastases are more likely to produce symptoms. Despite this assumption, our sensitivity analysis, which varied the difference in resectability between asymptomatic and symptomatic recurrence, led to a consistent ranking of the four strategies. True resectability is challenging to model, as it often involves interplay between the same disease burden characteristics, which can lead to symptoms, as well as surgical expertise to facilitate resection and any responses to perioperative systemic treatment [54]. Our study assumed consistent use of perioperative oxaliplatin-based chemotherapy for 6 months throughout our patient cohort [32]. This course of therapy has been demonstrated to improve disease-free survival but its impact on overall survival is uncertain, so it may not be routinely used in all real-world cases [55]. This assumption may have inflated the cost estimates associated with resectable recurrence, as previous models of metastasectomy did not account for the impact of systemic therapy [29].

The transition from asymptomatic to symptomatic recurrence in stage IV CRC was challenging to characterize, as it is not well described in the literature. We therefore performed a sensitivity analysis based on the only source identifiable for a possible range of possibilities [48]. The overall pattern of cost-effectiveness among the surveillance strategies did not change at the extremes of 1 month or 28 months to symptomatic recurrence. However, clinical judgement may play a role in modifying surveillance recommendations if there is sufficient evidence to suggest that a patient may have more indolent disease and a prolonged asymptomatic disease state.

This study is limited by various model assumptions. Modeling did not incorporate the impact of novel treatments (e.g., immunotherapy for high microsatellite instability, mismatch repair deficiency, or high tumor mutational burden cancers), or the addition of biological agents (e.g., panitumumab, cetuximab, and bevacizumab) or more nuanced prognostic factors (e.g., the presence of a *BRAF* mutation or sidedness of the primary tumor). Novel use of liquid biopsy for earlier stages of disease has also recently demonstrated benefits, though it is unclear what impact this may have on resected stage IV disease, but it may further refine risk stratification and surveillance. Given the era that the modelling data was constructed from, these details were not readily available for incorporation. Furthermore, many of these novel interventions have not been uniformly adopted in the real world. In addition, the existing risk-stratification Fong Clinical Risk Score was not utilized as it is only relevant for hepatic metastases, which represents a subset of our cases, and it reflects older approaches to metastatic resection where the focus was on the disease burden as opposed to the residual organ function required.

## 5. Conclusions

Surveillance can be an invaluable method for detecting recurrent disease in patients with stage IV CRC following curative-intent metastasectomy. In a Canadian context and based on NCCN guidelines, this cost-effectiveness analysis demonstrated that active surveillance with clinic visits, CEA testing, and CT every 12 months for 5 years were associated with the greatest cost-effectiveness when compared to alternative strategies with greater frequency of follow-up. Health economics considerations have an important role in guiding practice, but good clinical judgement is also required to optimize disease management in the individual patient. Further research is needed to assess adherence to surveillance guidelines and to confirm the impact of surveillance on patient outcomes in stage IV CRC.

## Figures and Tables

**Table 1:** Base-case input values for cost-effectiveness analysis of surveillance strategies for post-metastasectomy stage IV colorectal cancer.

Variable	Value	Spread *	Source(s)	Distribution Used in PSA
Cost variables				
Carcinoembryonic antigen test	60.62	NA	Alberta Medical Association [36]	NA
Clinic visit	25.94	10.37–41.60	Alberta Medical Association [37]	Gamma
Colonoscopy	1059.35	618.06–14,833.48	Heitman et al. [36]	Gamma
Computed tomography	300.15	NA	Government of Alberta [37]	Gamma
Hepatic metastasectomy	4086.72	741.75	McKay et al. [38]	Gamma
Post-anesthesia recovery	84.58	31.43	McKay et al. [38]	Gamma
Palliative chemotherapy	6409.77	NA	Yezefski et al. [39]	Gamma
Utilities				
No evidence of disease	0.78	0.23	Jeong et al. [40], Wiering et al. [41]	NA
Asymptomatic	0.68	0.28	Jeong et al. [40], Wiering et al. [41]	NA
Symptomatic	0.50	0.28	Jeong et al. [40], Miller et al. [42].	NA
Recurrence	0.74	0.25	Jeong et al. [40], Wiering et al. [41]	NA
Clinical variables				
Sensitivity				
Clinic visit	0.42	0.27–0.57	Kjeldsen et al. [43]	Beta
Carcinoembryonic antigen test	0.64	0.49–0.79	Tan et al. [44]	Beta
Computed tomography	0.83	0.68–0.98	Rose et al. [45]	Beta
Colonoscopy	0.95	0.80–1.00	Rose et al. [45]	Beta
Specificity				
Clinic visit	0.95	0.70–1.00	Kjeldsen et al. [43]	Beta
Carcinoembryonic antigen test	0.90	0.75–1.00	Tan et al. [44]	Beta
Computed tomography	0.93	0.78–1.00	Rose et al. [45]	Beta
Colonoscopy	1.00	0.85–1.00	Rose et al. [45]	Beta
Metastasectomy mortality	0.010	0.003–0.015	Ercolani et al. [46]	Beta
Undetected to symptomatic, months	4	1–28	Ackland et al. [47]	Gamma
Asymptomatic metastasectomy rate	0.23	NA	Lee-Ying et al. [15]	NA
Symptomatic metastasectomy rate	0.05	NA	Lee-Ying et al. [15]	NA

* Spread presented as range, standard deviation, or 95% confidence interval. PSA, probabilistic sensitivity analysis; NA, not applicable.

**Table 2:** Cost and utility of surveillance for a cohort of 10,000 patients after curative-intent metastasectomy of stage IV colorectal cancer over a 10-year period.

Surveillance Strategy	Cost, CAD	Incremental Cost, CAD	Effectiveness		Incremental Effectiveness		Incremental Cost-Effectiveness Ratio	
			QALY	LY	QALY Gained	LY Gained	CAD/QALY Gained	CAD/LY Gained
q12	7707	-	0.6797	0.8952	-		-	
q6/q12	14,175	6469	0.7453	0.9769	0.0656	0.0816	98,592	79,236
q6	14,191	16	0.7449	0.9763	−0.0004	−0.0006	Extended dominated ^†^	Extended dominated ^†^
q3/q12	19,890	5699	0.7692	1.0125	0.0243	0.0362	234,132	157,584

^†^ Dominated strategies were equally or less effective than a less costly strategy. QALY, quality-adjusted life-year; LY, life-year.

**Table 3:** Univariable sensitivity analyses of the impact of plausible variations in model parameters on incremental cost-effectiveness ratios of surveillance.

Variable	Incremental Cost-Effectiveness Ratio, CAD/QALY Gained		
	q6/q12 Strategy vs. q12 Strategy	q6 Strategy vs. q12 Strategy	q3/q12 Strategy vs. q12 Strategy
Variation in time to symptomatic recurrence, months			
1	78,436	Abs. dominated ^†^	92,246
28	527,497	Abs. dominated ^†^	396,203
Variation in the probability of repeat curative resection			
Decreased by 25%	112,263	Abs. dominated ^†^	238,812
Increased by 25%	88,414	Abs. dominated ^†^	187,704

^†^ An absolutely dominated strategy is less effective and more costly than comparator strategies.

## Data Availability

Data will not be shared as this study did not involve the use of new data.
